# IL-32 as a potential biomarker and therapeutic target in cardiovascular disease

**DOI:** 10.3389/fimmu.2026.1755759

**Published:** 2026-04-22

**Authors:** Lingyue Qiu, Jing Ye, Ling Liu, Qingwei Ji

**Affiliations:** 1 Guangxi Chest Pain Center, and Department of Cardiology, The People’s Hospital of Guangxi Zhuang Autonomous Region, Institute of Cardiovascular Diseases, Guangxi Academy of Medical Sciences, Nanning, China; 2 Department of Cardiology, Renmin Hospital of Wuhan University, Wuhan, China

**Keywords:** biomarker, cardiovascular diseases, cytokines, IL-32, inflammation

## Abstract

Cardiovascular diseases remain a leading cause of death worldwide, driven by complex pathological mechanisms involving various cytokines and inflammatory responses. IL-32, a recently discovered cytokine, has emerged as a key player in the pathogenesis of cardiovascular diseases, including atherosclerosis, myocardial infarction, and heart failure. This review summarizes the biological characteristics of IL-32 and its role in cardiovascular diseases, explores its involvement in inflammatory responses and other pathological processes, and evaluates its potential as both a biomarker and a therapeutic target. Collectively, this review aims to provide new insights for the prevention and treatment of cardiovascular diseases.

## Introduction

Cardiovascular disease (CVD) remains one of the leading causes of death worldwide, affecting billions of people and placing a tremendous burden on society and families (1). According to data from the World Health Organization, the mortality rate attributed to cardiovascular diseases ranks first among all-cause mortality, even surpassing that of tumors and strokes (2, 3). The development and progression of CVD are driven by multiple complex mechanisms, among which chronic inflammation is recognized as a key contributor (4). Studies have shown that chronic inflammatory states not only lead to vascular endothelial dysfunction but also trigger various pathological changes such as atherosclerosis and cardiac remodeling, significantly increasing the risk of cardiovascular events (5). Extensive evidence has shown that inflammatory mediators, including tumor necrosis factor-α (TNF-α), interleukin-6 (IL-6), and interleukin-1β (IL-1β), play central roles in cardiovascular pathology. In particular, the clinical importance of the IL-1 pathway was highlighted by the Canakinumab Anti-inflammatory Thrombosis Outcomes Study (CANTOS), in which IL-1β inhibition with canakinumab reduced recurrent cardiovascular events independently of lipid lowering (6–8). Interleukin-32 (IL-32) is a pro-inflammatory cytokine that has attracted increasing attention because of its ability to amplify inflammatory responses and modulate downstream signaling pathways associated with nuclear factor-κB (NF-κB), p38 mitogen-activated protein kinase (MAPK), and the production of other inflammatory mediators, including TNF-α, IL-1β, and IL-6 (4, 9). Emerging evidence suggests that IL-32 may participate in several key inflammatory cascades relevant to CVD, including those involved in atherosclerosis, myocardial infarction, hypertension, and heart failure. Therefore, a deeper understanding of the biological characteristics of IL-32 and its role in cardiovascular inflammation may provide new insights into its potential utility as a biomarker and therapeutic target in CVD.

## Biological characteristics of IL-32

First identified in 1992, IL-32 is a pleiotropic cytokine secreted by both immune and non-immune cells, particularly macrophages and CD4+ T lymphocytes (9). The expression of IL-32 is induced by various pro-inflammatory cytokines, which in turn promote the release of other cytokines such as TNF-α, IL-6, and IL-1β. This makes IL-32 play a significant role in the progression of various diseases, including autoimmune diseases and cancer (10–12).

Several isoforms of IL-32 have been identified, including IL-32α, IL-32β, IL-32γ, IL-32δ, IL-32ϵ, and IL-32θ (12, 13). The receptor for IL-32 has not been fully identified. Current research has found that cell membrane protease 3 (Proteinase 3, PR3), integrin αVβ3, and αVβ6 can all exert IL-32 receptor functions (13, 14). Through interactions with these proposed cell-surface partners, IL-32 has been implicated in the activation of downstream inflammatory pathways, including NF-κB and p38 MAPK signaling, as well as NOD-related pathways, thereby influencing the functions of multiple cell types, including macrophages and endothelial cells (12, 13, 15). Although these isomers share a similar core structure, they may act differently in various microenvironments, adding further complexity to the biological characteristics of IL-32 (10, 16).

Several IL-32 isoforms are generated by alternative splicing of the IL32 transcript, including IL-32α, IL-32β, IL-32γ, IL-32δ, IL-32ϵ, and IL-32θ (9). Although IL-32γ is the longest isoform and is often considered one of the most biologically active splice variants, the mechanisms governing extracellular release of IL-32 remain incompletely understood. While IL-32γ has been proposed to contain a sequence compatible with secretion, available evidence suggests that native IL-32 may also be released through a non-classical secretory pathway (17–19). Therefore, the presence of circulating or extracellular IL-32 may not be entirely dependent on a conventional signal peptide-mediated secretion process (15, 19). The different isoforms of IL-32 exhibit distinct functional differences. IL-32α promotes inflammation primarily through activation of the NF-κB pathway, whereas IL-32β is thought to exert tumor-suppressive effects in certain cancer types (20, 21). IL-32θ is an anti-inflammatory isoform of IL-32 that also exhibits tumor suppressor activity, highlighting its potential value in both inflammation-related disorders and cancer (22). Research has shown that IL-32γ can promote the release of various inflammatory factors, which is closely related to cardiovascular disease. **Table 1** summarizes the currently available evidence on the roles of IL-32 isoforms, as well as non-isoform-resolved IL-32 evidence, across major cardiovascular disease settings.

**Table 1:** IL-32 isoforms in cardiovascular diseases.

IL-32 isoform	Cardiovascular phenotype	Predominant role	Proposed mechanism	Supporting evidence	References
IL-32α	Atherosclerosis	Anti-inflammatory and anti-atherogenic	Suppresses endothelial inflammation and vascular smooth muscle cell activation; upregulates TIMP3 and RECK via suppression of miR-205, thereby limiting matrix remodeling and inflammatory signaling	Mechanistic cell and animal studies	(21, 29)
IL-32β	Atherosclerosis	Predominantly pro-inflammatory	Enhances endothelial inflammatory responses, including IL-6 induction and pathways linked to leukocyte recruitment and vascular dysfunction	Endothelial cell-based mechanistic studies	(27)
IL-32γ	Atherosclerosis	Predominantly pro-inflammatory, but context-dependent	Promotes endothelial activation and inflammatory signaling; may also modulate cholesterol efflux pathways through ABCA1, ABCG1, and LXRα-related signaling	Mechanistic studies in endothelial cells, hepatocytes, and experimental atherosclerosis models	(27, 32)
Not isoform-resolved	Hypertension/blood pressure dysregulation	Pro-inflammatory clinical association	Higher circulating IL-32 is associated with increased systolic blood pressure and impaired blood pressure control, consistent with a vascular inflammatory role	Human biomarker/clinical association study	(26)
Not isoform-resolved	Heart failure after myocardial infarction	Inflammation- and remodeling-associated	Elevated circulating IL-32 may reflect inflammatory activation, adverse remodeling, or pro-fibrotic signaling after MI	Prospective clinical study and *in vitro* fibroblast experiments	
Not isoform-resolved	SSc-associated pulmonary arterial hypertension	Pro-inflammatory/vascular injury-associated	Associated with vascular injury, endothelial proliferation, and inflammatory activation in SSc-associated PAH	Human biomarker study and *in vitro* endothelial experiments	(50, 53)

MI, myocardial infarction; PAH, pulmonary arterial hypertension; SSc, systemic sclerosis; TIMP3, tissue inhibitor of metalloproteinase 3; RECK, reversion-inducing cysteine-rich protein with Kazal motifs; LXRα, liver X receptor alpha.

## IL-32 and atherosclerosis, coronary artery diseases

Coronary artery disease (CAD) is characterized by narrowing or obstruction of the coronary arteries, leading to myocardial ischemia, with atherosclerosis serving as the primary underlying cause (23). Atherosclerosis is a chronic inflammatory disease characterized by endothelial damage, the deposition of lipids (especially oxidized low-density lipoprotein, ox-LDL) in the vascular wall, the proliferation of smooth muscle cells, and the formation of fibrous tissue, ultimately resulting in the narrowing of the arterial lumen and restricted blood flow (23–25). Circulating LDL-C can accumulate within the vascular wall and contribute to lipid plaque formation. When LDL is oxidized to ox-LDL, its detrimental effects on the vascular wall become even more pronounced. Ox-LDL promotes endothelial dysfunction, inflammatory responses, and macrophage uptake, thereby further accelerating the progression of atherosclerosis (24, 25). A substantial body of evidence from clinical studies indicates that regulating lipid metabolism significantly reduces the occurrence and progression of atherosclerotic diseases while improving patient prognosis (24).

Accumulating evidence suggests that IL-32 is involved in cholesterol metabolism (23, 26). Damen et al. found that IL-32 regulates cholesterol metabolism in the context of atherosclerosis. After stimulating human primary hepatocytes, the mRNA expression of IL-32α, IL-32β, and IL-32γ was significantly upregulated, and IL-32γ levels were strongly correlated with the cholesterol transport proteins ABCA1, ABCG1, the transcription factor LXRα, and apolipoprotein A1 (apoA1) (26). In the presence of endogenous IL-32, intracellular lipid concentrations were significantly reduced. Overexpression of IL-32γ significantly induced the expression of ABCA1, while silencing IL-32γ significantly reduced ABCA1 mRNA expression (26). However, IL-32α treatment led to decreased expression levels of ABCA1, LXRα, and PPARγ in THP-1 macrophages, enhanced lipid deposition in THP-1 macrophages stimulated with ox-LDL, and dose-dependently inhibited cholesterol efflux. These findings suggest that different isoforms of IL-32 have distinct, and even opposing, regulatory effects on cholesterol metabolism. IL-32 may also contribute to the pathological process of atherosclerosis by influencing the expression of cholesterol transport proteins, highlighting the diversity of IL-32 functions.

As inflammatory regulators, different IL-32 isoforms appear to play distinct roles in atherosclerosis. IL-32β and IL-32γ, as the major isoforms IL-32, significantly impact coronary artery endothelial cell (CAEC) function. Specifically, these isoforms upregulate endothelial adhesion molecules (e.g., ICAM-1 and VCAM-1) and chemokines (e.g., CCL-2 and CXCL-8), promoting monocyte migration, thereby exacerbating endothelial dysfunction and inflammatory responses (27). Furthermore, IL-32 expression correlates with arterial wall stiffness, suggesting it may play an important role in regulating the vascular wall, offering a new potential therapeutic target for atherosclerosis (28). However, Son et al. found that IL-32α regulates the Rprd2-Dgcr8/Ddx5-Dicer1 biosynthesis pathway by downregulating microRNA-205, which promotes the upregulation of the anti-atherosclerotic genes Timp3 and Reck. This in turn reduces inflammation-induced endothelial cell production of ICAM-1 and VCAM-1, inhibits the migration and proliferation of vascular smooth muscle cells (VSMCs), thereby exerting an anti-atherosclerotic effect (29). These studies suggest that different isoforms of IL-32 may play either protective or pathogenic roles in the process of atherosclerosis. IL-32 also appears to play an important role in the formation of atherosclerotic plaques. Studies have demonstrated that IL-32 expression is significantly elevated in the arterial plaques of atherosclerosis patients and is associated with plaque instability (28). IL-32 enhances plaque inflammatory responses by promoting the aggregation and activation of inflammatory cells, which may increase the risk of plaque rupture (30). Moreover, different IL-32 isoforms exhibit distinct biological effects in plaque formation and stability. IL-32β and IL-32γ are considered key pro-atherosclerotic factors, while IL-32α may exert protective effects under certain conditions (17).

**Figure 1:**
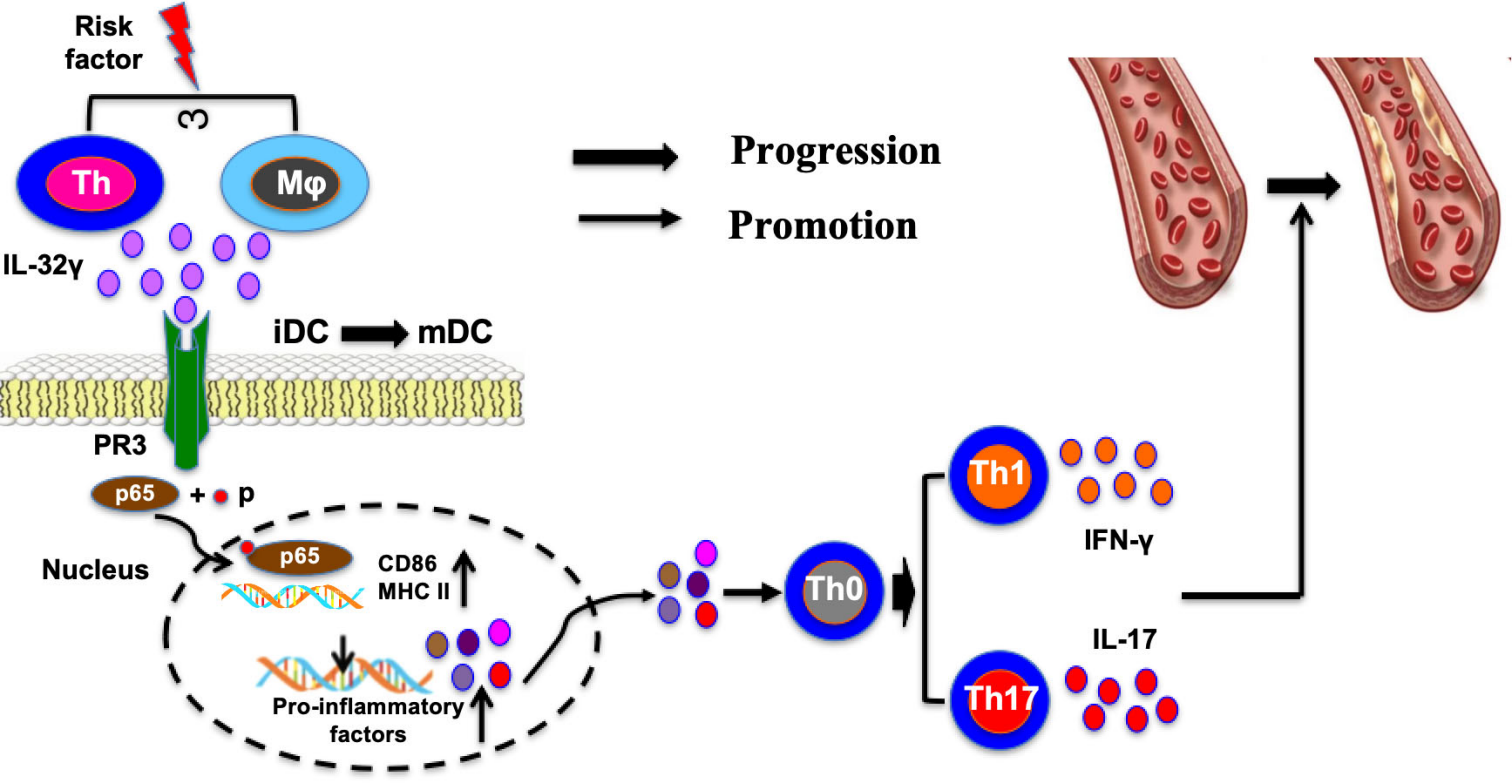
Schematic summary of the proposed mechanisms linking IL-32γ to atherosclerosis progression. Atherosclerosis-related inflammatory stimuli may increase IL-32γ expression. IL-32γ is proposed to interact with proteinase 3 (PR3) and activate NF-κB signaling, thereby promoting the maturation of dendritic cells from immature dendritic cells (iDCs) to mature dendritic cells (mDCs). Mature dendritic cells may further enhance adaptive immune activation and promote Th1/Th17-associated inflammatory responses, leading to increased secretion of inflammatory cytokines such as IFN-γ and IL-17. In the cardiovascular context, IL-32 may also affect other vascular-relevant cell types, including endothelial cells, macrophages, and vascular smooth muscle cells, thereby contributing to endothelial dysfunction, inflammatory amplification, and plaque progression. This figure summarizes currently available evidence and should be interpreted as an integrative mechanistic model rather than a definitive pathway.

Numerous studies have shown that the levels of IL-32 in the circulation and plaques of CAD patients are significantly elevated and closely associated with the severity of the disease (31). In one study, coronary artery specimens from six individuals with normal coronary arteries and eight patients with CAD undergoing heart transplantation were analyzed by immunofluorescence staining. IL-32 expression was significantly higher in atherosclerotic plaques than in normal coronary arteries and was detected in macrophages, CD4+ T cells, vascular smooth muscle cells (VSMCs), and endothelial cells (ECs), with macrophages and CD4+ T cells representing the predominant IL-32-expressing cell populations. Compared to the normal coronary artery (NCAD) group, the CAD group showed significantly increased levels of IL-32, IFN-γ, and IL-17, which progressively increased in the stable angina pectoris (SAP), unstable angina pectoris (UAP), and acute myocardial infarction (AMI) groups. Additionally, plasma IL-32 levels in CAD patients were positively correlated with IFN-γ, IL-17, and the Gensini score. Linear regression analysis indicated that IL-32 was independently associated with the occurrence of CAD. Recently, we demonstrated that exogenous IL-32γ exacerbates atherosclerosis progression in ApoE−/− mice. In that study, IL-32γ was upregulated in patients with atherosclerosis, while *in vitro* experiments showed that ox-LDL induced IL-32γ expression in endothelial cells through NF-κB activation. Endothelial-derived IL-32γ promoted macrophage migration and M1 polarization through the p38 MAPK pathway. *In vivo*, IL-32γ enhanced plaque formation and macrophage infiltration in ApoE−/− mice, whereas these effects were attenuated by p38 inhibition (32). Collectively, these findings suggest that IL-32γ may act as an important mediator linking endothelial dysfunction to vascular inflammation during atherosclerosis. The potential mechanisms by which IL-32γ contributes to atherosclerosis progression, including dendritic cell activation and broader vascular inflammatory responses, are summarized schematically in **Figure 1**.

Several single-nucleotide polymorphisms (SNPs) in the *IL32* gene, including rs28372698 and rs4786370, have been associated with susceptibility to CAD. Studies have found that the homozygous TT genotype and T allele of rs28372698 are associated with an increased risk of CAD, while the TT homozygote and T allele of rs4786370 are associated with a decreased CAD risk (33–35). However, these two SNPs have no significant effect on IL-32 levels or the severity of coronary artery stenosis. While IL-32 has been implicated in CAD, the precise genetic mechanisms underlying its involvement warrant further investigation (35). Collectively, these observations point to a critical role for IL-32 in the pathogenesis of CAD.

Myocardial infarction is an acute and life-threatening complication of coronary artery disease. The larger the area of myocardial infarction, the worse the patient’s prognosis. Experimental studies suggest that IL-32 may exacerbate myocardial infarction (36). Researchers used a mouse myocardial infarction model by ligating the left anterior descending artery (LAD) and injected recombinant human IL-32α and IL-32γ into four sites around the infarction border zone. Results indicated that recombinant IL-32 significantly increased the infarction size in the mouse myocardial infarction model. Cellular experiments showed that recombinant IL-32 promotes the expression of MMP-9, and TGF-β in cardiac fibroblasts, which may represent one of the mechanisms underlying the enlargement of myocardial infarct size. These findings suggest that IL-32 may contribute to myocardial injury and may represent a potential therapeutic target for limiting infarct size.

The role of IL-32 in myocardial ischemia-reperfusion injury (MIRI) has garnered increasing attention (37). Available evidence suggests that IL-32 levels increase during ischemia-reperfusion and are involved in the regulation of oxidative stress, inflammation, and apoptosis. Mechanistically, IL-32 activates the NOD2/NOX2/MAPK signaling pathway, promoting oxidative stress and inflammatory responses in cardiomyocytes, which in turn leads to cardiomyocyte apoptosis and functional impairment (37). Elucidating this process may provide new therapeutic targets for post-myocardial infarction management, as inhibition of IL-32 expression could help alleviate myocardial damage and improve prognosis.

## IL-32, hypertension, pregnancy-induced hypertension, and pulmonary hypertension

**Figure 2:**
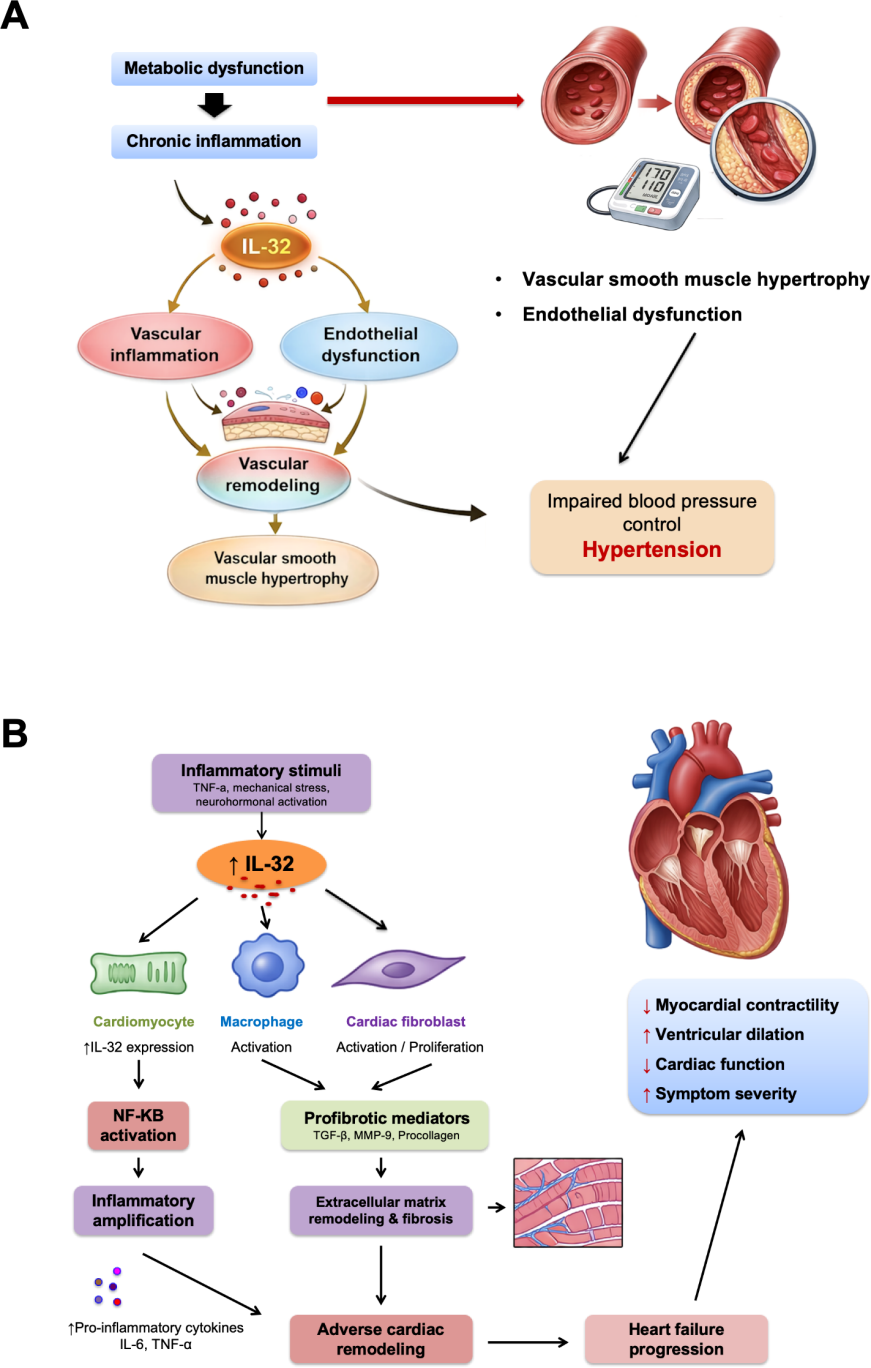
**(A)** Proposed mechanisms linking IL-32 to hypertensive vascular dysfunction.Chronic low-grade inflammation associated with metabolic dysfunction may increase IL-32 expression. Elevated IL-32 may contribute to vascular inflammation, endothelial dysfunction, and vascular remodeling, thereby impairing blood pressure control and promoting hypertension. In the cardiovascular context, these processes may be accompanied by vascular smooth muscle hypertrophy and structural vascular changes. This figure summarizes currently available evidence and should be interpreted as a proposed mechanistic model rather than a definitive pathway. **(B)** Proposed mechanisms linking IL-32 to heart failure progression. Inflammatory stimuli, particularly TNF-α, may induce IL-32 expression in cardiomyocytes through activation of NF-κB signaling. Increased IL-32 may amplify inflammatory responses and promote activation of cardiac fibroblasts, leading to increased expression of profibrotic and matrix-remodeling mediators, including TGF-β, MMP-9, and procollagens. These processes may contribute to extracellular matrix remodeling, interstitial fibrosis, adverse cardiac remodeling, and progressive deterioration of cardiac function. This figure represents an integrative summary of currently available evidence.

Hypertension is one of the most common chronic diseases worldwide and a major risk factor for cardiovascular diseases. Metabolic syndrome (MetS) refers to a cluster of cardiovascular and diabetes-related risk factors, including abdominal obesity, hyperglycemia, hyperlipidemia, and hypertension (38). In recent years, growing evidence has revealed that inflammation serves as a bridge linking metabolic syndrome and hypertension, both of which are closely associated with chronic low-grade inflammation. Common inflammatory factors such as TNF-α, IL-6, and CRP play important roles in the pathogenesis of both conditions (39). Metabolic syndrome may exacerbate the development of hypertension, whereas hypertension can further worsen the clinical manifestations of metabolic syndrome, forming a vicious cycle (40–42). A study from the Liver-Bible-2021 cohort, which included 949 patients with metabolic dysfunction, showed that plasma IL-32 levels were significantly elevated in patients with both metabolic dysfunction and hypertension and were positively correlated with systolic blood pressure (43). The study also revealed that in patients receiving antihypertensive treatment, IL-32 levels significantly decreased, suggesting that antihypertensive drugs may suppress the release of IL-32 by improving blood pressure control. These findings further support a potential role for IL-32 in blood pressure regulation, although the precise mechanisms underlying this effect remain to be elucidated. The potential mechanisms by which IL-32 may participate in hypertensive vascular dysfunction are summarized schematically in **Figure 2A**.

Pregnancy-induced Hypertension (PIH) is a common pregnancy complication associated with multiple factors, particularly inflammatory factors involved in its onset and progression. PIH encompasses conditions such as gestational hypertension and preeclampsia (PE), which can affect the placenta, maternal vascular system, and fetal health. The placenta contains various trophoblast cell types, such as syncytiotrophoblasts, villous cytotrophoblasts, and extra villous trophoblasts (EVTs) (21, 44, 45). Trophoblast invasion is essential for the complete function of the placenta, and insufficient or abnormal invasion can lead to preeclampsia, intrauterine growth restriction, and PIH (44, 46, 47). Cytokines produced by trophoblasts or other cellular components play important roles in regulating trophoblast invasion. Studies have shown that IL-32 enhances trophoblast cell migration and invasion in a dose-dependent manner, accompanied by increased IL-32 mRNA expression (21). Mechanistic studies revealed that IL-32 upregulates the expression of miR-205, which in turn enhances IL-32 expression, forming a positive feedback loop. Furthermore, IL-32 promotes trophoblast invasion via the NFκB-MMP2/9 axis, contributing to the pathogenesis of PIH. Interestingly, the study also found that in PIH placentas, IL-32 and miR-205 levels were significantly lower compared to normal pregnancies. Collectively, these findings suggest that a better understanding of the regulatory network involving IL-32 and miR-205 may provide insight into the prevention and treatment of PIH.

Systemic Sclerosis (SSc) is a chronic, systemic autoimmune disease with widespread clinical manifestations, often accompanied by fibrosis of the blood vessels, skin, and internal organs (48). Both hypertension and pulmonary hypertension (PH) are common complications in SSc patients, with pulmonary hypertension being particularly hazardous. Studies have shown that immune dysregulation in SSc leads to systemic inflammatory responses, promoting immune cell infiltration and the release of immune factors (such as interleukins and tumor necrosis factors), which in turn exacerbate inflammatory damage and vascular remodeling in the pulmonary vasculature (5, 42, 49). This process leads to narrowing and occlusion of pulmonary arteries and arterioles, ultimately resulting in pulmonary arterial hypertension (PAH), one of the most common causes of death in SSc patients. Due to the nonspecific symptoms of PAH, diagnosis is often delayed, making the improvement of existing diagnostic methods and the development of new evaluation strategies particularly important (48). Research has shown that serum IL-32 levels in patients with SSc combined with pulmonary arterial hypertension (SSc-PAH) are significantly higher than in SSc patients without PH and in idiopathic pulmonary arterial hypertension (iPAH) patients. ROC curve analysis demonstrated that a serum IL-32 cutoff of 11.12 pg/ml effectively predicted pulmonary hypertension in patients with 90% sensitivity and 100% specificity (50, 51). Additionally, a significant correlation was found between IL-32 serum levels, and both mean pulmonary arterial pressure (mPAP) and systolic pulmonary arterial pressure (sPAP) in SSc patients. Researchers have also observed elevated IL-32 levels in the skin of patients with SSc-PAH. Nold et al. found that in normal individuals, IL-32 is primarily present in vascular smooth muscle cells of the pulmonary small arteries, while in PAH, endothelial cells (ECs), particularly hyperproliferative ECs, exhibit higher IL-32 expression and contribute to the obstruction of the vascular lumen. *In vitro* experiments showed that when endothelial cells were stimulated by VEGF receptor inhibitors, IL-32 expression was significantly increased. Silencing IL-32 using siRNA technology revealed that the reduction of IL-32 could significantly inhibit the proliferation of endothelial cells under IL-1β stimulation (14, 16, 52). Moreover, exogenous stimulation of IL-32, under IFN-γ pre-treatment, also enhanced the proliferative capacity of endothelial cells. These findings suggest that aberrant IL-32 expression may promote endothelial proliferation, contribute to vascular lumen obstruction, and participate in the progression of PAH (53).

## IL-32 and heart failure

Heart failure is a complex disease caused by multiple factors. With the increasing prevalence of cardiovascular disease and an aging population, the incidence and mortality rates of heart failure continue to rise. CAD, hypertension, and cardiomyopathy are the primary causes of heart failure (54–56). In recent years, chronic inflammation has been considered a key factor in the onset, progression, and prognosis of heart failure. As cardiac function declines, dysfunction in the heart and multiple systemic organs triggers the release of inflammatory mediators, including TNF-α, IL-6, and TGF-β (56–58). These factors not only participate in immune responses but also exacerbate heart failure by promoting processes such as myocardial hypertrophy, fibrosis, and necrosis. Inflammatory cytokines are involved not only in the development of heart failure but also closely associated with poor prognosis in heart failure patients and serve as biomarkers for predicting outcomes (59). Elevated levels of inflammatory markers are often indicative of worse clinical outcomes, including higher mortality and hospitalization rates. Therefore, modulating the inflammatory response may represent a therapeutic strategy to improve prognosis in heart failure patients.

In a cellular experiment, researchers treated cardiomyocytes with different doses of TNF-α, revealing a close relationship between IL-32 and TNF-α during the progression of heart failure (58). The results showed that as the dose increased, cardiomyocytes exhibited significantly reduced contractile function, increased cell death rates, and markedly elevated markers of apoptosis and necrosis (60). Gene Ontology (GO) enrichment analysis indicated that TNF-α induced a robust inflammatory response in cardiomyocytes, with multiple pro-inflammatory cytokines upregulated, among which IL-32 showed the most significant elevation. IL-32 was found to be involved in T cell-mediated chronic inflammatory responses. Mechanistic studies showed that TNF-α binds to cardiomyocytes via its receptor TNFR1, activating the NF-κB signaling pathway, which promotes IL-32 expression. Given that IL-32 is also an inducer of TNF-α, these findings suggest that a positive feedback loop involving TNF-α and IL-32 may amplify inflammatory responses and further impair cardiac function during the progression of heart failure.

A prospective clinical study involving 100 patients with chronic heart failure secondary to myocardial infarction showed that baseline plasma IL-32 levels significantly increased with higher NYHA functional class. IL-32 levels were negatively correlated with LVEF and positively correlated with heart failure biomarker NT-proBNP, myocardial fibrosis markers Procollagen type III, and MMP9 (36). Over a median follow-up of 1.8 years, high IL-32 levels significantly increased the risk of all-cause mortality or heart failure hospitalization. Autopsy results of heart failure cases showed that cardiomyocytes are the primary source of IL-32 in cardiac tissue. *In vitro* cell experiments further confirmed that treatment with recombinant IL-32α increased the activity of cardiac fibroblasts, upregulating the expression of MMP-9, PI, PIII, and TGF-β. Collectively, these findings indicate that IL-32 not only serves as a prognostic biomarker in heart failure but also actively contributes to the progression of myocardial fibrosis. The proposed mechanisms linking IL-32 to inflammatory amplification, fibrosis, and adverse remodeling in heart failure are summarized in **Figure 2B**.

Dilated cardiomyopathy (DCM) is another common cause of heart failure, characterized by left ventricular dilation and impaired systolic function (1, 61). Increasing evidence suggests that the extensive network of inflammatory cells and cytokines plays a critical role in the pathological process of DCM (62–64). A study explored the relationship between *IL-32* gene polymorphisms and susceptibility to DCM in the Chinese Han population. The research team recruited 418 patients diagnosed with DCM and 437 healthy controls. Genotyping was performed for two SNPs, rs12934561 and rs28372698, using the polymerase chain reaction-restriction fragment length polymorphism (PCR-RFLP) method (34). The results showed that, compared to the healthy control group, the C allele and CC genotype of rs12934561 were significantly elevated in DCM patients, while the A allele and AA genotype of rs28372698 were significantly reduced. This suggests that *IL-32* gene polymorphisms may be associated with DCM risk, with rs12934561 potentially serving as a predictive marker for screening high-risk populations. These findings suggest that IL-32 polymorphisms may be associated with susceptibility to DCM. However, given the case-control design and the lack of functional validation, the clinical significance of these variants and their potential utility for risk stratification require further investigation. The currently available evidence on IL-32 polymorphisms and cardiovascular-related phenotypes is summarized in **Table 2**.

**Table 2:** Summary of evidence on IL-32 polymorphisms and cardiovascular-related phenotypes.

SNP	Population	Phenotype/outcome	Risk-associated allele/genotype	Main findings	Functional implication
rs28372698	Chinese Han patients undergoing coronary angiography (175 CAD, 56 non-obstructive CAD, 131 controls)	CAD susceptibility	T/TT	TT genotype and T allele were associated with a higher risk of CAD.	No significant association with plasma IL-32 levels or Gensini score.
rs4786370	Chinese Han patients undergoing coronary angiography (175 CAD, 56 non-obstructive CAD, 131 controls)	CAD susceptibility	T/TT as a protective direction	TT genotype and T allele were associated with a lower risk of CAD.	No significant association with plasma IL-32 levels or angiographic severity.
rs12934561	Southwest Chinese Han population (418 DCM, 437 controls)	DCM susceptibility	C/CC	C allele and CC genotype were associated with an increased risk of DCM.	Association appeared more evident in the low-LVEF subgroup.
rs28372698	Southwest Chinese Han population (418 DCM, 437 controls)	DCM susceptibility	T as a risk direction; A/AA as a protective direction	A allele and AA genotype were less frequent in DCM, suggesting a protective association of A.	Functional significance was not directly examined.
rs28372698	Swedish elderly community cohort (486 participants, mean age 77 years, 7.1-year follow-up)	All-cause and cardiovascular mortality	A/AA	AA genotype was associated with higher all-cause and cardiovascular mortality after multivariable adjustment.	No association with plasma IL-32; circulating IL-32 did not predict cardiovascular mortality.
rs4786370	General population cohort plus two RA cohorts	Lipid intermediate phenotypes (mainly HDL-C)	C/CC as a potentially favorable direction	CC genotype was associated with higher HDL-C; associations with LDL-C and total cholesterol were less consistent.	Suggests a possible role in inflammatory regulation and lipid metabolism.

Risk-associated allele/genotype refers to the direction of association for the specific endpoint in each individual study, rather than a universally established causal variant. Because the included studies addressed disease susceptibility, prognosis, and intermediate phenotypes, their findings should not be directly compared across endpoints.

## Therapeutic prospects of targeting IL-32

IL-32 has emerged as a potential therapeutic target because of its broad involvement in inflammatory amplification and tissue remodeling. Potential strategies include direct neutralization using blocking antibodies, suppression of IL-32 expression by siRNA or other gene-silencing approaches, and indirect inhibition through disruption of IL-32-related signaling pathways or receptor interactions. Preclinical studies in non-cardiovascular inflammatory models have shown that anti-IL-32 antibodies, IL-32-specific siRNA, and blockade of IL-32 binding to proteinase 3 can attenuate downstream inflammatory responses (65). However, in cardiovascular disease, therapeutic targeting of IL-32 remains largely at the preclinical stage, and further studies are needed to clarify isoform-specific effects, safety, and translational feasibility. This limited translational progress likely reflects the absence of a fully established canonical receptor, the existence of multiple isoforms with potentially distinct or even opposing effects, and the lack of cardiovascular disease-specific therapeutic studies.

## Conclusion

Current evidence indicates that IL-32 not only plays a role in regulating inflammatory responses but may also directly influence cardiovascular health by affecting processes such as cholesterol metabolism, cardiomyocyte apoptosis, endothelial function, and atherosclerosis. Multiple studies have confirmed that IL-32 expression levels are closely associated with various cardiovascular diseases, such as hypertension, CAD, and heart failure, suggesting that IL-32 could serve as a potential biomarker and therapeutic target. Although IL-32 has shown promise as a biomarker in several cardiovascular-related settings, its sensitivity and specificity have not been systematically established across disease categories. Larger, well-designed studies with standardized assays and validated cutoff values are needed before IL-32 can be considered a reliable cardiovascular biomarker.

However, despite studies revealing the association between IL-32 and cardiovascular diseases, there remain some discrepancies among findings. Some studies suggest that IL-32 may have protective effects, while others indicate that it may promote cardiovascular pathological processes. These divergent views may stem from differences in study design, sample selection, and experimental conditions.

Overall, the role of IL-32 in cardiovascular disease remains incompletely understood. Future studies should address several key questions, including the isoform-specific effects of IL-32, its tissue- and cell-specific expression patterns, the characterization of its receptor and downstream signaling pathways, and the validation of circulating IL-32 as a biomarker in larger, well-defined clinical cohorts. In addition, future translational studies should determine whether therapeutic targeting of IL-32 can achieve disease-specific benefits without disrupting its potentially context-dependent or protective effects. Clarifying these issues will be essential for determining the translational value of IL-32 in cardiovascular medicine.
